# Challenges in antibody structure prediction

**DOI:** 10.1080/19420862.2023.2175319

**Published:** 2023-02-12

**Authors:** Monica L. Fernández-Quintero, Janik Kokot, Franz Waibl, Anna-Lena M. Fischer, Patrick K. Quoika, Charlotte M. Deane, Klaus R. Liedl

**Affiliations:** a Department of General, Inorganic and Theoretical Chemistry, University of Innsbruck, Innsbruck, Austria; b Center for Protein Assemblies (CPA), Physics Department, Chair of Theoretical Biophysics, Technical University of Munich, Garching, Germany; c Department of Statistics, University of Oxford, Oxford, UK

**Keywords:** Antibodies, antibody structure, protein structure prediction, biophysical surface properties, structural inaccuracies

## Abstract

Advances in structural biology and the exponential increase in the amount of high-quality experimental structural data available in the Protein Data Bank has motivated numerous studies to tackle the grand challenge of predicting protein structures. In 2020 AlphaFold2 revolutionized the field using a combination of artificial intelligence and the evolutionary information contained in multiple sequence alignments. Antibodies are one of the most important classes of biotherapeutic proteins. Accurate structure models are a prerequisite to advance biophysical property predictions and consequently antibody design. Specialized tools used to predict antibody structures based on different principles have profited from current advances in protein structure prediction based on artificial intelligence. Here, we emphasize the importance of reliable protein structure models and highlight the enormous advances in the field, but we also aim to increase awareness that protein structure models, and in particular antibody models, may suffer from structural inaccuracies, namely incorrect cis-amide bonds, wrong stereochemistry or clashes. We show that these inaccuracies affect biophysical property predictions such as surface hydrophobicity. Thus, we stress the importance of carefully reviewing protein structure models before investing further computing power and setting up experiments. To facilitate the assessment of model quality, we provide a tool “TopModel” to validate structure models.

## Breakthroughs in protein/antibody structure prediction

Predicting the three-dimensional (3D) structure of a protein based solely on the amino-acid sequence is one of the grand challenges in the field of protein structure prediction.^1^ Accurate prediction of the 3D structure of a protein is critical to understand its function, as the shape of the protein determines its properties and ultimately its function. To determine the state-of-the-art methods in protein structure prediction, the biennial community-based benchmarking experiment “Critical Assessment of methods in protein Structure Prediction (CASP)” was established.^2–4^ In CASP14 (2020), DeepMind showcased AlphaFold2, a program based on artificial intelligence (AI) that directly processes multiple sequence alignments.^5^ Comparable accuracies in predicting protein structures can also be achieved with other methods including RoseTTAFold,^6^ and specialized tools for antibodies which incorporate the recent advances.^7–9^ Those tools are highly accurate based on global measures, often with root mean square deviations (RMSDs) to the crystal structure of less than 1 Å. However, there are often higher inaccuracies in specific parts of the protein that should be carefully reviewed.^10,11^ Post-translational modifications are omitted, but can sometimes be added afterwards.^12^ Furthermore, the accuracy for multimers, such as antibodies, is still lower.^13^ Additional challenges can arise for antibodies since VDJ recombination events do not follow the classical pathway of evolution.^14^

Antibodies are crucial components of the adaptive immune response.^15^ Genetic recombination and somatic hypermutation events enable the adaptive immune system to produce a vast number of antibodies against a variety of pathogens.^14^ To understand and optimize antigen recognition and to enable rational design of antibodies, accurate structure models are essential.^16^ Despite these recent advances, accurate structure prediction of antibodies remains challenging and still needs to be extensively validated. In particular, the flexible loops involved in recognizing the antigen pose a major challenge.^17,18^ In comparison to other protein superfamilies, the fold of antibodies is generally highly conserved.^19–21^ In particular, the framework of the antigen-binding fragment (Fab) is structurally almost identical for all antibodies.^22,23^ However, the area hardest to predict accurately is the six hypervariable loops that can form, together with several framework residues, the antigen-binding site, engaging with the respective epitope. These loops are also known as the complementarity-determining region (CDR) and provide the sequence and structure diversity essential to recognize a wide range of antigens. Five of the six loops tend to adopt canonical cluster folds based on their length and sequence composition. However, the third CDR loop of the heavy chain, the CDR-H3 loop, is the most diverse in length, sequence and structure and therefore is the most challenging loop to predict accurately.

Various methodologies have been developed to improve and advance antibody structure prediction, by incorporating different homology model strategies or using the canonical cluster model,^24–28^ but recently various antibody-specific deep learning methods such as ABlooper, DeepAb and IgFold have significantly improved the CDR loop modeling accuracy.^7–9,29^ The predicted structure models achieve similar or better quality than methods that are able to predict all types of protein structures (including AlphaFold2).^13,30^ Additionally, the CDR-H3 loop conformation is also strongly influenced by the relative interdomain orientation, as it is located in the center, directly in the interface between the heavy and the light chains. It has been shown that, in addition to the CDR loops, the relative interdomain orientation plays a crucial role in defining the shape of the antigen binding site.^31–33^ Therefore, accurate predictions of the relative V_H_-V_L_ interdomain orientations are vital to characterize the topology of the antigen binding site.^24^ To measure the relative V_H_-V_L_ interdomain orientation, various approaches have been used, the most widely known being ABangle.^32^ All these improvements have enabled predictions of a vast number of antibody structures at a high level of accuracy, which can then further be used as input structures for virtual screening or to inform rational design of antibodies.^16,17^

## Possible inaccuracies in antibody structure models

**Figure 1.**
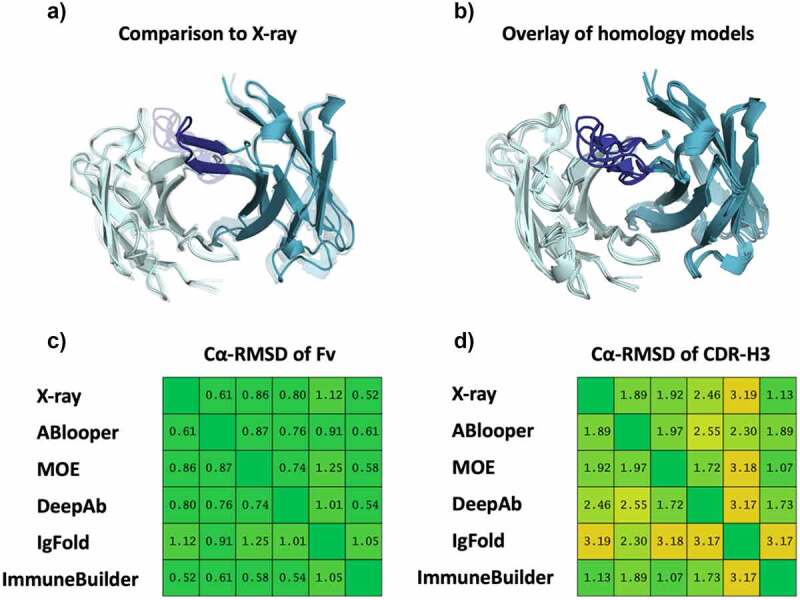
A) Comparison of the available X-ray structure of the CIS43 antibody (PDB code: 7SG5) with the structure models generated with different antibody prediction tools, namely ABlooper, MOE, DeepAb, IgFold and ImmuneBuilder. B) Structural overlay of the obtained Fv models, showing the high variability in the CDR-H3 loop. C) Cα-RMSD matrix of the X-ray structure and the respective models for the whole Fv. D) Cα-RMSD matrix of the X-ray structure and the respective models for the CDR-H3 loop.

Here, we investigated a dataset consisting of 137 antibody sequences published by Jain et al.^34^ and used the available antibody structure prediction tools ABlooper,^7^ IgFold,^8^ DeepAb,^9^ Immunebuilder,^29^and the MOE Antibody Modeler^35^ to generate structure models for further biophysical characterizations.^36,37^ Careful inspection of the generated models revealed inconsistencies such as cis-amide bonds in the CDR loops, D-amino acids and severe clashes. In total, this resulted in up to 300 D-amino acids and up to 240 cis-amide bonds for the 137 antibody models. We found cis-amide bonds and clashes independent of the applied antibody modeling tools. The only tools that did not introduce any D-amino acids are DeepAb and Immunebuilder. The recently available Immunebuilder tool performs physical plausibility checks, i.e., checks for steric clashes, cis-amide bonds, or bonds with nonphysical lengths. These structural inaccuracies affect the results of structure-based biophysical property predictions.^36,37^ In addition to the Jain et al. dataset,^34^ we predicted the structure of the CIS43 antibody, where the experimental X-ray structure (PDB accession code: 7SG5)^38^ was released after the structure prediction tools were published, to have an experimental reference structure outside of the used training set. Figure 1 shows an overlay of all obtained structure models and reveals an overall high structural similarity, reflected in low overall RMSD values (~1 Å). However, substantial structural variability can be observed in the CDR-H3 loop (RMSD values >2 Å). This result points out one particular challenge in predicting antibody structures: the high variability of the antibody CDR loops cannot be captured or represented by a single static structure.^39–41^ While there can be properties and metrics that are not too much affected by these issues, metrics that rely on accurate CDR-H3 structures will be strongly distorted. This includes antibody-antigen docking, since the CDR-H3 loop is a central part of the binding interface, as well as structure-based hydrophobicity calculations, since the binding interface frequently contains more hydrophobic amino acids than the rest of the antibody surface.^16,17,36^ Molecular dynamics (MD) simulations might correctly capture the ensemble in solution if the starting structure is sufficiently close, but current MD approaches cannot sample cis-trans isomerization or transitions from D- to L-amino acids, leading to ensembles that are potentially worse than the starting structure in terms of RMSD to the crystal structure (SI Figure S1 and S2). Additionally, SI Figure S2 shows the relative interdomain orientations of the CIS43 antibody obtained by MD simulations with the orientations of the models (originating from different tools) projected as vertical lines. For this example, the models differ in their interdomain orientation up to 5° from the available X-ray structure. However, all of the orientations found in models preexist in the MD ensemble distribution.

**Figure 2.**
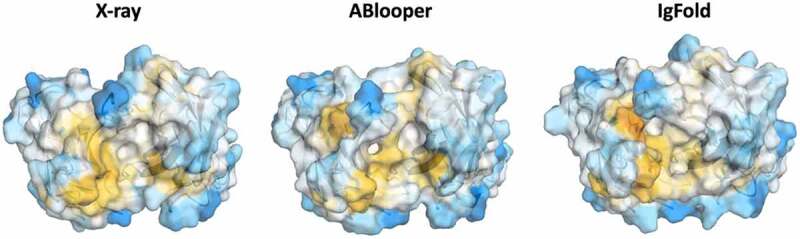
Surface hydrophobicity mapped on the X-ray structure and two antibody models. Surface hydrophobicity was assigned by the Whimley and White hydrophobicity scale. Hydrophobic areas are colored in yellow, while hydrophilic parts are depicted in blue.

To show the effect of starting structures with cis-amide bonds and D-amino acids in the CDR loops, we compared the surface hydrophobicity of structure models for the CIS43 antibody variant with the X-ray structure (Figure 2). The surface hydrophobicity was assigned using the hydrophobicity scale by Wimley and White.^42^ We found differences in the surface hydrophobicity, which is expected as hydrophobicity is potentially a strongly conformation-dependent property, since small sidechain rearrangements may expose otherwise buried hydrophobic groups. While small inaccuracies in the atomic positions can be fixed by MD simulations, the correct sidechain packing is often impossible when D-amino acids or cis-amide bonds are present, leading to almost irreparable errors in the biophysical property estimation. The same is true for antibody-antigen docking, where an accurate representation of the surface is required to find the correct interactions with the antigen.

**Figure 3.**
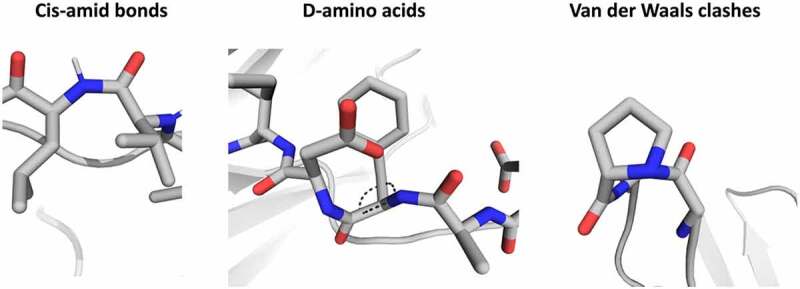
Examples of structural inaccuracies observed in some of the models, namely cis-amide bonds, D-amino acids and Van der Waals clashes.

Figure 3 shows examples of cis-amide bonds and D-amino acids in the CDR-H3 loop of CIS43.

Additionally, in one of the obtained models we found a missing proline sidechain at the tip of the CDR-H3 loop. Rebuilding the proline results in severe clashes, as shown in the right panel of Figure 3, and the only way to avoid these clashes would be to build a D-Proline instead.

**Figure 4.**
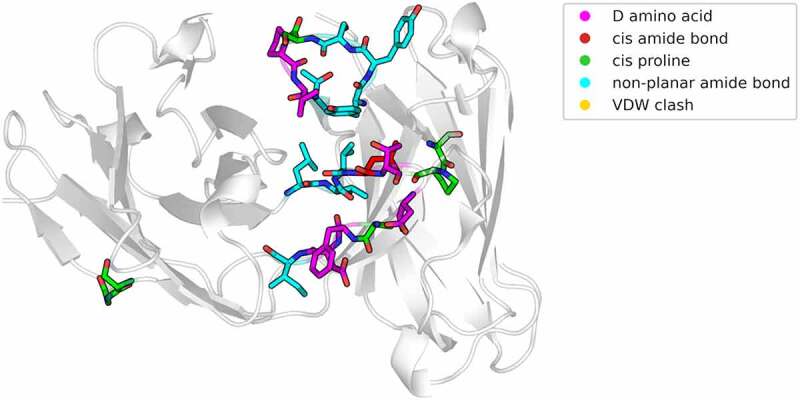
Pymol visualization of the structural inaccuracies, as output of the “TopModel” tool.

To facilitate the identification of these issues, we present a tool (available on Github: https://github.com/liedllab/TopModel), called “TopModel”, that quickly checks the structure for cis-amide bonds, D-amino acids, and clashes. With this tool, structure models can rapidly be checked to assess the quality and accuracy of the models before performing further analysis. At the same time, it offers the possibility to directly visualize these issues in PyMOL^43^.Figure 4 shows the output obtained by “TopModel”. Residues colored in magenta (D-amino acids) and red (cis-amide bonds) represent issues that should be fixed, while cis-Prolines are colored green, as they occasionally occur in native protein structures. Van der Waals clashes are colored in yellow. For the clashes we also provide an optional score, to quantify the quality of the model, that takes the number of clashes and the length of the protein into account, to quantify the quality of the model. Non-planar amide bonds are depicted in cyan. As these issues can also be found for other protein structure models apart from antibodies, we recommend checking every model with “TopModel” and stress the importance of validating the obtained structures to ensure the most accurate results/predictions as possible.

## Discussion

While we want to highlight the enormous advances in the protein structure prediction field, at the same time, we must also emphasize the importance of critically reviewing structure models, especially antibody models, before basing conclusions, further experiments, or MD simulations on potentially erroneous models.^44^ In particular, we stress the importance of not limiting the characterization of the antigen binding site to a single-static structure model, as there is already high structural variability between the different models (Figure 1). This high variability suggests that even considering ensembles in solution might more accurately reflect the properties and functions of the antibody (SI Figure S1-S3).^18^ The tremendous development in protein structure prediction enables fast protein structure predictions that approach the accuracy of experimental structures.^30^ These breakthroughs have been achieved by combining artificial intelligence with an effective exploitation of the available structural information and incorporation of evolutionary related sequences through multiple sequence alignments (MSAs).^30^ AlphaFold, RoseTTAFold, ESMFold and other methods have revolutionized protein structure prediction.^6,30,45–47^ Various deep learning (based) approaches have also been shown to improve antibody structure prediction and outperform all previously available antibody structure prediction methods.^7–9,48^ Accurately predicting the structure of antibodies is central to understand their function, to elucidate antibody-antigen binding and inform rational antibody design.

However, the most challenging part in antibody structures prediction is concentrated in the six CDR loops, as they reveal the highest variability in both sequence and structure.^15,26^ In particular, the CDR-H3 loop reveals the highest diversity and variability, which impedes the accurate prediction of its structure.^40,49^ This is in line with our findings, as the comparison of different antibody prediction methods reveals the most diverging results for the CDR-H3 loop. Figure 1 shows an overlay of all the obtained models, highlighting the high conformational variability of the CDR-H3 loop. To account for this high diversity of the CDR loops one single static structure might not be sufficient and therefore the CDR loops should rather be characterized as ensembles in solution.^18,40^ Another critical aspect in antibody structure prediction is the relative interdomain orientation (V_H_-V_L_).^32^ The orientation of the two variable domains, V_H_ and V_L_, strongly influences the shape of the antigen binding site and consequently plays a role in antigen recognition.^31,50^ A comparison of the different structure models of the CIS43 antibody reveals differences in the interdomain orientations of up to 5°, which can be captured already with short MD simulations (SI Figure S3).^50,51^ Thus, these findings suggest, that already short MD simulations can be sufficient to optimize the interdomain orientation.^50^

This is especially important, as various biophysical properties of antibodies, such as hydrophobicity, are conformation dependent and already small sidechain rearrangements reveal distinct surface properties.^36,37^ MD simulations provide such ensembles in solution, increasing the probability that conformations determining biophysical properties are captured.^36^ In agreement with these observations, we find that these conformational differences between the models can result in changes in the surface hydrophobicity.

In addition to the high divergence in CDR-H3 loop conformations, we found various cis-amide bonds, D-amino acids, and clashes in the obtained models. Such modeling artifacts are non-natural and can strongly influence biophysical property predictions, resulting in misleading conclusions (SI Figure S1).^44^ Thus, to address these pitfalls, we provide a tool that quickly inspects protein structure models and identifies issues and flaws in the protein structures, namely the python package “TopModel”. As accurate antibody structures are a prerequisite to reliably understand antibody function and characterize biophysical properties, we strongly suggest an additional validation of the respective structure models to increase the quality of the respective predictions.

## Methods

The tool “TopModel” (version 1.0) inspects and highlights issues in a structure model. “TopModel” checks the chirality, amide bond stereochemistry and Van der Waals clashes for every residue in the structure model. The structure models are parsed and analyzed using biopython.^52^ To calculate the chirality a triangle is defined based on the priority of the atom chains around the chiral center. The direction of a normal vector to this plane, calculated using the cross product, is determined by the priority of the atom chains. By calculating the dot product of this normal vector and a vector from the chiral center to the plane, the orientation of the three atom chains with respect to the center can be determined. Based on the sign of the resulting scalar, the chirality can be assigned. This approach allows us to calculate the chirality even if no hydrogens are included in the structure model. The amide bond is inspected by calculating the dihedral angle of the protein backbone. Cis-amide bonds to proline are labeled separately as they naturally occur more frequently than in other amino acids. Dihedral angles that could neither be assigned cis nor trans are labeled as non-planar. The Van der Waals clashes are quickly computed using a k-d tree^53^ and Van der Waals radii data gathered using the python package Mendeleev.^54^ For the clashes an optional score can be displayed, which takes the number of clashes and the length of the protein into account. The chiralities and amide bond orientations are not included in the score. To quickly assess the structural implications of the issues found by “TopModel”, the analyzed structure can be opened in PyMOL^43^ with the issues highlighted and labeled as shown in Figure 4.

## Supplementary Material

Supplemental Material

## Figures and Tables

**Table:** 

CASP	Critical Assessment of methods in protein Structure Prediction
CDR	Complementarity Determining Region
Fab	Antigen-binding fragment
Fv	Variable fragment
MSA	Multiple Sequence Alignment
NMR	Nuclear Magnetic Resonance
PCA	Principle Component Analysis
PDB	Protein Data Bank
RMSD	Root Mean Square Deviation
