# Supplementary material for: Challenges in antibody structure prediction
Source: MAbs. 2023 Feb 12;15(1):2175319. doi: 10.1080/19420862.2023.2175319 (PMC9928471; doi:10.1080/19420862.2023.2175319)
Supplement: Supplemental Material [file KMAB_A_2175319_SM9100.docx]

Supporting Information:


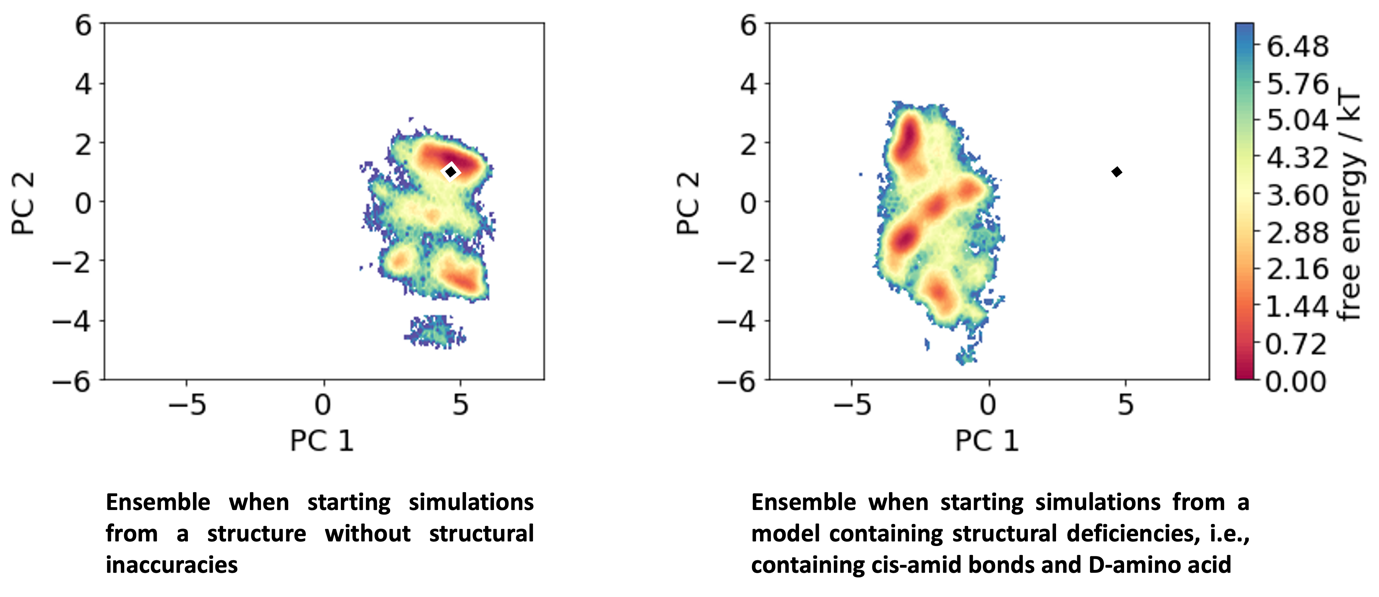


SI Figure S1: Principal Component Analysis (PCA) comparing an MD ensemble in solution of all six CDR loops starting from a structure without structural inaccuracies (left) with an ensemble obtained using a structure with deficiencies. The diamond projected into the PCA space represents the available X-ray structure for the CIS43 antibody (PDB accession code: 7SG5).


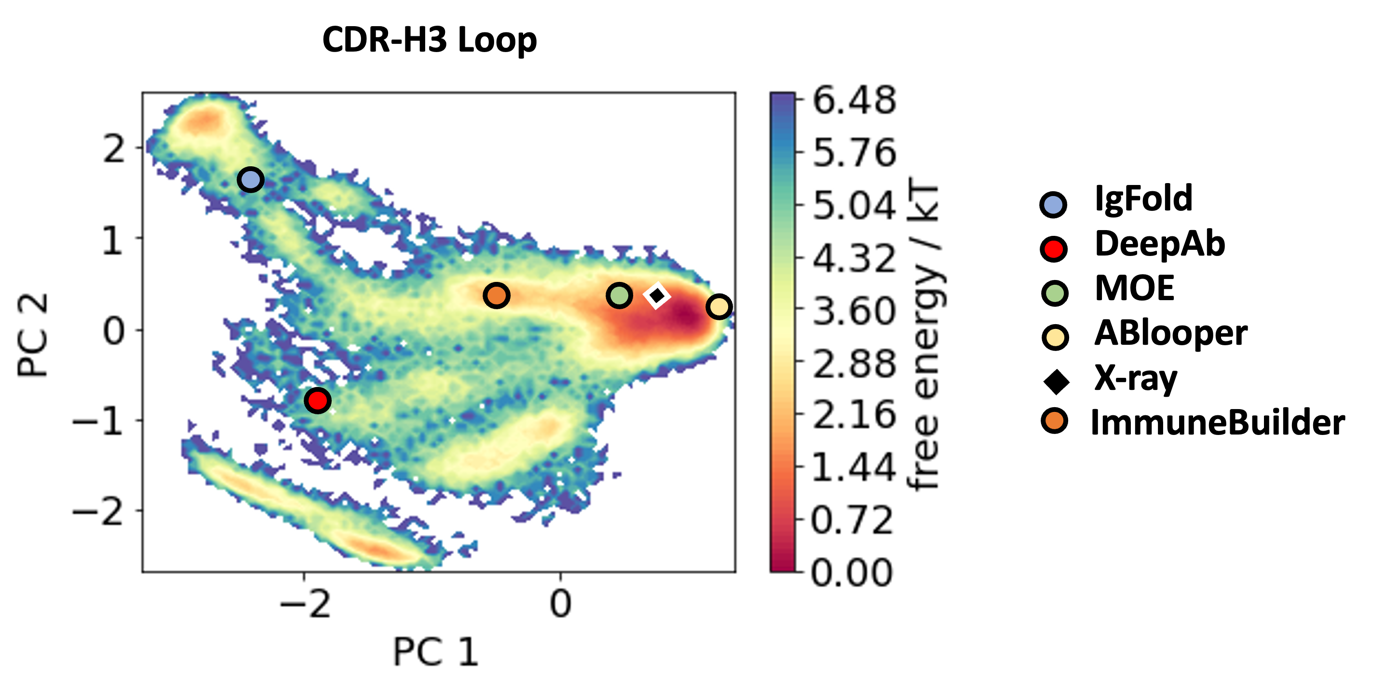


SI Figure S2: Principal Component Analysis (PCA) of the CDR-H3 loop ensemble in solution including the projected structure models, color-coded circles, and the X-ray structure, as black diamond.


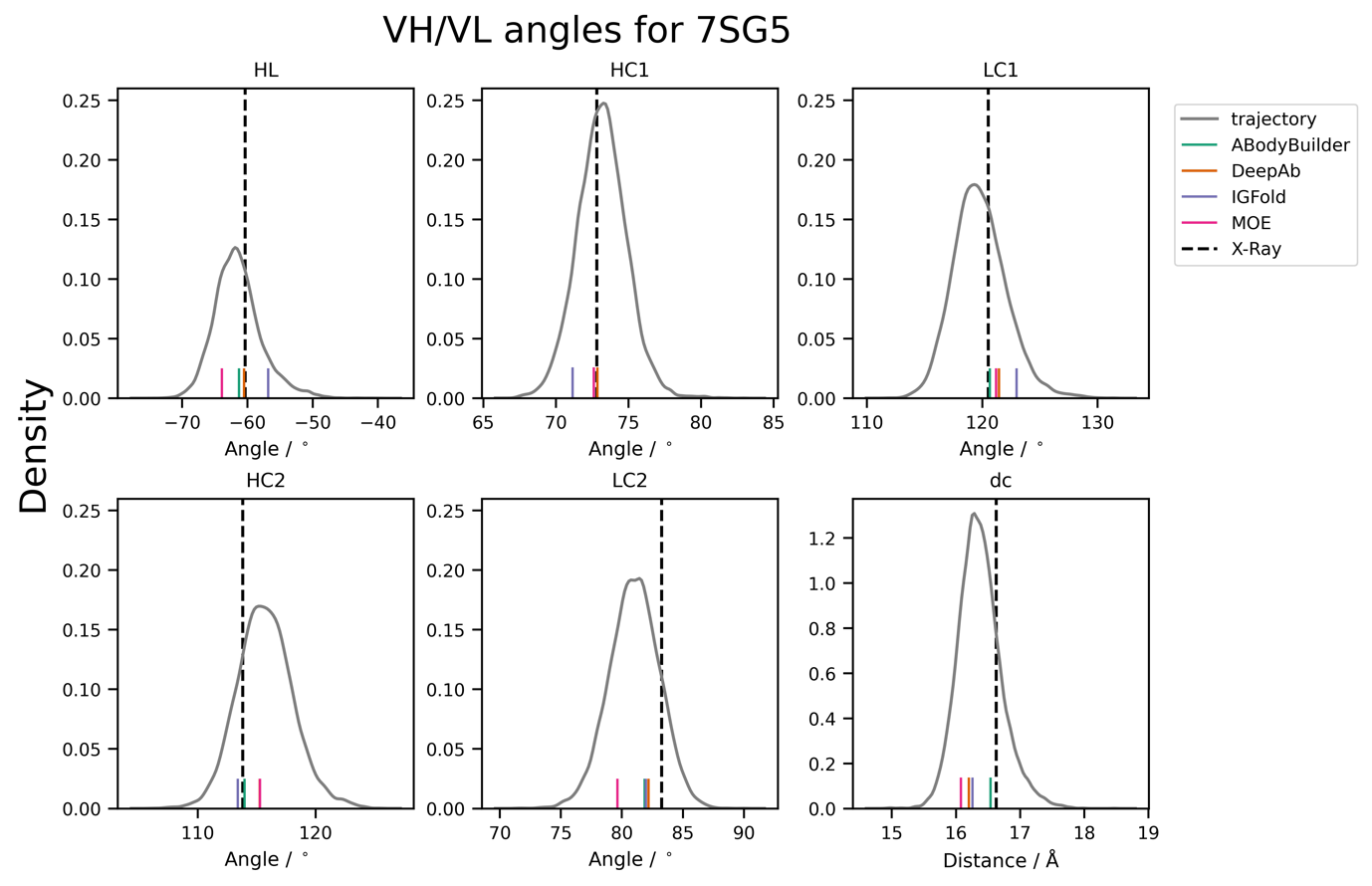


SI Figure S3: Interdomain angle/distance distributions obtained from an MD ensemble in solution including the respective interdomain orientations from the models, projected as colored vertical lines.

Alt Text:

SI Figure S1: Two free energy landscapes of the CIS43 antibody in the same coordinate system showing areas with higher energies in blue and with lower energies in red. No conformational overlap can be identified for the ensembles started from a structure with physical inaccuracies and the ensemble obtained using the X-ray structure as starting point.

SI Figure S2: Free energy landscape of the CDR-H3 loop of the CIS43 antibody showing again areas with high energies in blue and with lower energies in red. Additionally, differently colored dots are projected into the conformational space, representing the different structure models compared in this study.

SI Figure S3: Six interdomain angle and distance distributions obtained from MD simulations for the CIS43 antibody are represented as grey line spanning an angle range of about 20° and a distance range of about 2 Å. The projected vertical colored lines represent the different structure models and lie within the MD interdomain angle distributions, differing in up to 5° from the available X-ray structure interdomain orientation.
